# Silicate of Potash

**Published:** 1876-02

**Authors:** 


Silicate of Potash in the Treatment of Erysipelas.—P. F. da Costa Alvarenza recommends that this fluid should be applied once or several times daily with a camel-hair brush to the affected part, either in the pure state or diluted in various proportions with water, as 1 to 4 or 1 to 11. The more concentrated the solution the more rapid the cure. The author has applied it to forty-eight cases in Lisbon and in seven at Rio Janeiro, and considers it to be the most effective as well as the most economical remedy that can be employed. According to Ullersperger it has been already employed to a considerable extent in Germany. Especially also as an injection in catarrh of the bladder.—*Cent., f. d. Chir.*

